# Two-Front War on Cancer—Targeting TAM Receptors in Solid Tumour Therapy

**DOI:** 10.3390/cancers14102488

**Published:** 2022-05-18

**Authors:** Agata Mikolajczyk, Filip Mitula, Delfina Popiel, Bozena Kaminska, Maciej Wieczorek, Jerzy Pieczykolan

**Affiliations:** 1 Preclinical Development Department, Celon Pharma S.A., Research & Development Centre, Marymoncka 15, 05-152 Kazun Nowy, Poland; delfina.popiel@celonpharma.com (D.P.); jerzy.pieczykolan@celonpharma.com (J.P.); 2 Postgraduate School of Molecular Medicine, Medical University of Warsaw, 02-091 Warsaw, Poland; b.kaminska@nencki.edu.pl; 3 Clinical Development Department, Celon Pharma S.A., Research & Development Centre, Marymoncka 15, 05-152 Kazun Nowy, Poland; maciej.wieczorek@celonpharma.com

**Keywords:** MERTK, AXL, TYRO3, TAM family, receptor tyrosine kinase, targeted therapy, cancer

## Abstract

**Simple Summary:**

In recent years, many studies have shown the importance of TAM kinases in both normal and neoplastic cells. In this review, we present and discuss the role of the TAM family (AXL, MERTK, TYRO3) of receptor tyrosine kinases (RTKs) as a dual target in cancer, due to their intrinsic roles in tumour cell survival, migration, chemoresistance, and their immunosuppressive roles in the tumour microenvironment. This review presents the potential of TAMs as emerging therapeutic targets in cancer treatment, focusing on the distinct structures of TAM receptor tyrosine kinases. We analyse and compare different strategies of TAM inhibition, for a full perspective of current and future battlefields in the war with cancer.

**Abstract:**

Receptor tyrosine kinases (RTKs) are transmembrane receptors that bind growth factors and cytokines and contain a regulated kinase activity within their cytoplasmic domain. RTKs play an important role in signal transduction in both normal and malignant cells, and their encoding genes belong to the most frequently affected genes in cancer cells. The TAM family proteins (TYRO3, AXL, and MERTK) are involved in diverse biological processes: immune regulation, clearance of apoptotic cells, platelet aggregation, cell proliferation, survival, and migration. Recent studies show that TAMs share overlapping functions in tumorigenesis and suppression of antitumour immunity. MERTK and AXL operate in innate immune cells to suppress inflammatory responses and promote an immunosuppressive tumour microenvironment, while AXL expression correlates with epithelial-to-mesenchymal transition, metastasis, and motility in tumours. Therefore, TAM RTKs represent a dual target in cancer due to their intrinsic roles in tumour cell survival, migration, chemoresistance, and their immunosuppressive roles in the tumour microenvironment (TME). In this review, we discuss the potential of TAMs as emerging therapeutic targets in cancer treatment. We critically assess and compare current approaches to target TAM RTKs in solid tumours and the development of new inhibitors for both extra- and intracellular domains of TAM receptor kinases.

## 1. Introduction

The TAM receptor family comprises three receptor tyrosine kinases (MERTK, AXL, TYRO3) that play important roles in diverse biological processes in normal cells [1,2,3,4]. The tyrosine kinases MERTK, AXL, and TYRO3 share a typical RTK structure with an extracellular domain (ECD) of two immunoglobulin-related domains (IgL), followed by two fibronectin type III (FNIII), a transmembrane domain, and a tyrosine kinase domain (TKD) on the cytoplasmic side of the membrane [5,6,7]. The human TAMs exhibit 31–36% identical (52–57% similar) amino acids (aa) within the extracellular region and the intracellular domains share 54–59% aa identity (72–75% similarity) within the tyrosine kinase domain [6].

TAM receptors are activated upon binding their extracellular ligands with IgL domains, subsequent receptor dimerisation, cross-autoactivation of TKD, and downstream signal transduction [8,9]. The first natural ligands of TAM receptors, i.e., Growth Arrest-Specific 6 (GAS6) and Protein S (PROS1), were identified in 1995 and to date, they remain the best-known ligands of the TAM family [7,10,11]. GAS6 and PROS1 require vitamin K-dependent γ-carboxylation for maximal activation. The ligands have a distinct affinity for the different TAM receptors: GAS6 binds all TAM receptors, with the highest affinity for AXL, while protein S binds to MERTK and TYRO3. GAS6 and PROS1 can bind the phosphatidylserine (PS), and TAMs become activated when PS is exposed on the apoptotic cells, aggregating platelets, exosomes, or virus envelopes [1,12,13,14].

The activated TAM receptors stimulate many downstream signalling pathways, including the phosphatidylinositol-3-kinase (PI3K)/Akt and the mammalian target of rapamycin (mTOR) PI3K-Akt-mTOR, the MAPK/ERK kinase (MEK) 1/2-extracellular signal-regulated kinase (ERK) MEK-ERK, p38 MAPK, FAK (focal adhesion kinase), STAT (signal transducer and activator of transcription), and NFκB (nuclear factor-κB), impacting cell proliferation, migration, survival, and epithelial–mesenchymal transition (EMT) [15,16,17,18,19,20].

The TAM RTK are typically not mutated in cancer cells; however, their overexpression has been reported in numerous solid tumours and hematologic malignancies, including non-small cell lung cancer (NSCLC), glioblastoma, melanoma, prostate cancer, breast cancer, acute myeloid leukaemia, and others [21,22,23,24,25,26,27]. The expression of AXL corresponds with a metastatic propensity, invasiveness in vitro, and resistance to targeted therapies [28,29,30,31]. AXL and MERTK proteins have been increasingly implicated in drug resistance to both conventional and targeted therapies [17,32,33,34]. Moreover, the association of EMT with AXL kinase expression is well documented, and EMT in turn is highly correlated with drug resistance [35,36,37]. A growing number of findings show the involvement of the activated TAM kinases in cancer progression [15,21,24,35,38].

The immunoregulatory roles of MERTK RTK were demonstrated in single- or multiple TAM knockout mice where a lack of these receptors resulted in the development of a severe lymphoproliferative disorder along with autoimmunity [39]. The most-studied effects of TAM knockout have been in the immune system, where signalling from the receptors couples the clearance of cell debris with the negative regulation of the innate immune system [40,41,42]. One of the striking features of TAM single, double, and triple knockout mice were connected with autoimmunity and impaired apoptotic cell clearance. What is worth mentioning is that the inhibition of more than one TAM family kinase resulted in a more severe phenotype [39,43,44].

Both MERTK and AXL enhance the immunosuppressive nature of the tumour niche, as they are expressed on macrophages, NK cells, and dendritic cells [45,46,47,48]. It has been shown that the TAM receptors expressed in the tumour microenvironment play a role in the phagocytosis of apoptotic cells, differentiation of NK cells, the function of T cells, and the secretion of cytokines [24,46,49,50,51].

**Figure 1:**
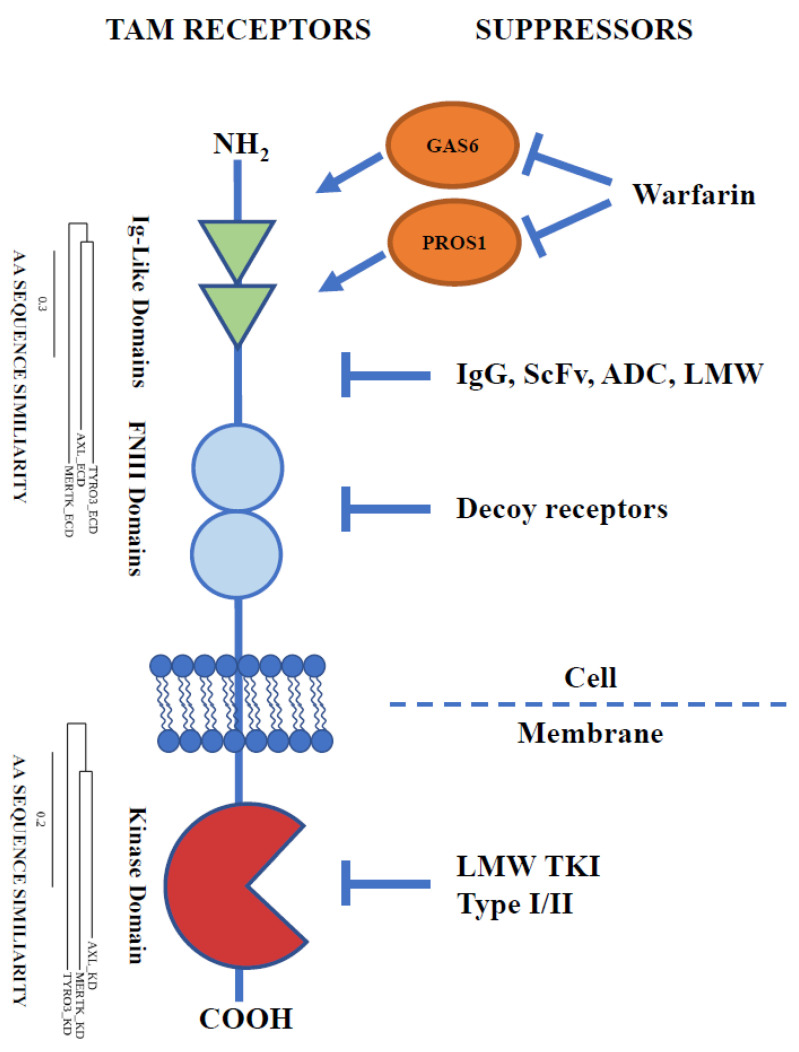
Different strategies of inhibition of TAM receptor activity. Abbreviations: GAS6, Growth arrest-specific 6 ligand; PROS1, Protein S; IgG, immunoglobulin G antibodies; scFv, single-chain variable fragment antibodies; ADC, antibody–drug conjugates; LMW, low-molecular-weight compounds; TKI, tyrosine kinase inhibitors; IgG-like domains, immunoglobulin-like domains; FNIII domains, fibronectin type III domains; AA, amino acid; ECD, extracellular domain; KD, kinase domain.

In recent years, TAM kinases have become an important therapeutic target in cancer therapy (Figure 1). Due to the similarities in the structure of the three TAM receptors, it is difficult to develop inhibitors specific to a single TAM RTK. Therefore, there are currently three main strategies implemented to inhibit TAM receptor activity and signal transduction in carcinogenesis: (1) inhibition of ligand–receptor complex formation; (2) decoy receptors in a soluble form designed to form inactive TAM complexes; and (3) small-molecule tyrosine kinase inhibitors (TKIs). In this review, we discuss how these strategies are applied to battle carcinogenesis, how successful they are, and provide support for the further drug development of TAM-targeting therapies.

## 2. TAM Family in Carcinogenesis

The classical activation of RTKs involves ligand binding to the extracellular domain of the protein [52]. Subsequently, ligand binding causes receptor dimerisation and the subsequent autophosphorylation of tyrosine residues within the cytoplasmic domain [53]. Recent studies demonstrated that each TAM receptor has a distinct pattern of activation by GAS6 and PROS1, and their interactions may be affected by the presence of apoptotic cells and PS-containing lipid vesicles [9]. Tsou et al. showed that the γ-carboxylation of ligands was required for the full activation of TAMs and soluble immunoglobulin-like TAM domains could act as specific ligand antagonists [9]. Although TYRO3, AXL, and MERTK share sequence similarity, they have distinct functions in the immunoregulation and the recognition/removal of apoptotic cells [9]. In two models of TAM-dependent homeostatic phagocytosis, MERTK played a dominant role, while AXL was dispensable; the activation of MERTK by PROS1 was sufficient to drive phagocytosis [54].

Ligand binding induces receptor dimerisation and subsequent trans-autophosphorylation of tyrosine residues within the cytoplasmic domain and activation of adaptor signalling proteins, which results in the initiation of the signalling cascade and phosphorylation of the downstream targets [55]. TAM family members share three conserved tyrosine residues within the activation loop in the kinase domain. Those sites are required for proper kinase enzymatic activity, and in the human, the conserved tyrosines are as follows: Tyr698, Tyr702, Tyr703 in AXL, Tyr749, Tyr753, and Tyr754 in MERTK, and Tyr681, Tyr685, and Tyr686 in TYRO3 [56,57]. Other phosphorylation sites of TAM proteins are positioned in the distal part of the cytoplasmic domain: Tyr779, Tyr821, and Tyr866 in AXL, Tyr847, Tyr872, and Tyr929 in MERTK, and Tyr762, Tyr804, and Tyr828 in TYRO3 [58,59].

In addition to ligand-dependent activation, the receptor-independent activation of TAM kinases also occurs and encompasses receptor-independent homodimerisation or aggregation of extracellular AXL domains on neighbouring cells [60,61,62]. Heterodimerisation between TAM family proteins has also been reported [63,64,65]. Moreover, some researchers have described heterodimerisation with non-TAM family receptors, such as EGFR or HER3, which activates AXL-associated signalling [28,66].

Various factors regulate the activity and expression of TAM kinases at different levels [67]. TAM kinases can be upregulated or downregulated at the transcriptional level, among others, through the action of cytokines. Post-transcriptional regulation also occurs via micro-RNAs, for example, miR-34a and miR-199a/b regulate AXL expression [68,69]. At the protein level, a metalloproteinase A disintegrin, and metalloprotease (ADAM)10 or ADAM17, may shed the extracellular domain of these proteins [70,71,72]. In addition, soluble forms of TAM receptors can inhibit the activity of these kinases by acting as a decoy receptor for ligands and preventing kinase stimulation by, for instance, GAS6 [73,74]. Although TAM family kinases are frequently overexpressed and activated in various types of cancer, genetic changes within their encoding genes are rather rare. Due to impaired phagocytosis, known relevant mutations in rodents and humans in *MERTK* genes could lead to *retinitis pigmentosa* [75,76]. In cancer, mutations, fusions, or amplifications in the TAM coding genes are not very common. However, the *AXL* aberrations have been identified in 3% or less of solid cancers (breast cancer, lung cancers, head, and neck cancer) and acute myeloid leukaemia [77,78].

## 3. Extracellular Domain: An Approach from the Outside

### 3.1. Ligand Binding/Dimerisation Inhibition

GAS6 and PROS1 are vitamin K-dependent proteins and share ~44% similar structural homology [79]. The general structure of GAS6 and PROS1 consists of a gamma-carboxy glutamic acid (Gla) domain at the N-terminus, then four epidermal growth factor-like (EGF-like) repeats, and at the C-terminus, a sex hormone-binding globulin (SHBG) domain made of two laminin G-like (LG) domains [80,81]. The Gla domain in the presence of vitamin K is γ-carboxylated and, in this form, recognises phosphatidylserine presented on the surface of apoptotic cells, which next forms a bridge between TAM receptors and an apoptotic cell [9]. The LG domains within the carboxy-terminal SHBG domains of the ligands are recognised and bound by IgL domains in TAM ECD. Upon ligand binding, the dimerisation of receptors occurs and is mediated by membrane-proximal fibronectin type III (FNIII) domains [8].

Although GAS6 and PROS1 are quite structurally similar, the functional differences and the distinct affinities of TAM receptors for these ligands are well established. GAS6 can bind to any of the TAM receptors, with the highest affinity for AXL, then TYRO3 and MERTK [9,54,82]. Interestingly, GAS6 has two binding sites reported—the major one is recognised exclusively by the AXL protein, whereas the minor GAS6 binding site is recognised by MERTK and TYRO3 [8]. This selectivity results from a β-sheet formation of charged and neutral residues within the major binding site of GAS6 to opposite faces of the newly formed β-sheet [8]. PROS1 presents a different binding profile, with a preference for TYRO3 and MERTK over AXL [83,84,85]. GAS6 and PROS1 could also be recognised by TAMs in dimeric forms, as there are reports of the formation of heterodimers GAS6-PROS1 and ligand multimerisation required for TAM receptor activation in certain scenarios [86,87].

A fine balance of ligand interaction with a tyrosine kinase receptor is necessary to maintain normal tyrosine kinase function without causing overactivation, which could result in human disease [88]. GAS6 is the most important ligand in anticancer therapy targeting TAM receptors. It is expressed in many human tissues and different types of cancers, and by binding to its three receptors (AXL, MERTK, TYRO3) plays a role in biological processes, i.e., proliferation, apoptosis, migration, and survival [89]. Notably, GAS6 expression within solid and non-solid tumours often correlates with poor prognosis [90,91,92,93]. Interestingly, this protein also plays a role in the TME [47,94,95]. References to overexpression of PROS1 in neoplasms are, so far, only single reports. PROS1 is known for its involvement in coagulation processes due to the thrombin-sensitive region within its structure, correlating with the blood coagulation cascade [96,97].

Antagonistic antibodies are a common strategy used to inhibit TAM signalling, due to their high specificity and versatility in blocking receptor–ligand complex formation. Monoclonal antibodies (mAbs) have been employed to stop not only simple ligand binding, but also TAM downstream activity. The formation of the antibody–receptor complex might also lead to the blockage of the receptor dimerisation, which may further result in receptor destabilisation and subsequent degradation. An immunological aspect of antibodies cannot be overlooked, as it may lead to the death of a cancer cell. Several mAbs have been designed for ECD of AXL, with proven activity against TAM receptors in vitro and in vivo. One, Tilvestamab, significantly inhibits AXL activation and tumour growth in mice [98] and is currently being tested in clinical trials (NCT04893551). Other antibodies did not reach this stage, but still present proven activity against TAM receptors in vitro and in vivo. Antibodies YW327.6S2, MAb173, D9/E8, 20G7-D9, 3G9/8B5/12A11/4F8 mAbs, and DAXL-88 block GAS6 binding to the receptors and reduce TAM transcription levels, with prominent inhibition of tumour growth, cell migration, and invasion in several cancer cell lines: SKOV3 (ovarian cancer), A549 (non-small cell lung cancer), and MDA-MB-231 (triple-negative breast cancer) [99,100,101,102,103]. Notably, there was an attempt to create a tetravalent bispecific IgG-scFv antibody format, combining anti-AXL CDX-0168 and anti-PD-L1 mAb (9H9) using an IgG-scFv format. In vitro results proved that this construct inhibited both PD-L1 and AXL signalling, as well as improved cytokine release and T-cell activation [104]. CDX-0168/9H9 was not further investigated and did not move to the clinical stage.

The antibody hTyro3-IgG against TYRO3 has been reported to induce drug sensitivity in primary colon cancer cell cultures and mouse xenografts [105]. RGX-019 humanised monoclonal antibody promotes MERTK receptor internalisation. MERTK signalling pathway inhibition reduced cancer cell viability and induced cytokine expression in the immune-suppressive M2 macrophages. The RGX-019 antibody also showed a good profile in toxicity studies, as it did not reveal retinal toxicity, a common undesirable effect of MERTK inhibition [106].

Although only one of these antibodies reached the clinical stage of development, antibody–drug conjugates (ADC) were more successful in this field and brought a definitive cytotoxic effect to highly specific anti-AXL antibodies. Two different ADCs are currently in clinical trials. Mecbotamab vedotin (CAB-AXL-ADC) is in the I/II clinical phase in patients with advanced solid tumours in phase 1 and BA3011 alone, and with a PD-1 inhibitor in phase II (clinical trials NCT03425279 and NCT04681131). Enapotamab vedotin (HuMax^®^-AXL-ADC) has shown some clinical activity in phase II for solid tumours, but was discontinued in 2020, as the data gathered during the trials did not meet the desired criteria (NCT02988817).

### 3.2. Receptor Cleavage and Decoy Receptors

RTKs in certain cellular conditions can be cleaved by ADAM10/17 to release the kinase domain into the cytoplasm [70,71,72]. TAM’s proteolytic cleavage is increased by lipopolysaccharide (LPS), phorbol 12-myristate 13-acetate (PMA), reactive oxygen species (ROS), and other environmental factors [71]. This mechanism can be exploited in cancer treatment, as TAM ECD in the soluble form is rendered dysfunctional, therefore it cannot transduce signal downstream; moreover, it may inhibit intact TAM receptor signalling by interacting with TAM ligands to limit their accessibility.

Interestingly, while soluble forms of TYRO3 and AXL effectively blocked both PROS1 and GAS6 signalling, respectively, soluble MERTK showed weak inhibitory activities against both ligands [9]. Still, the soluble form of MERTK (sMer) was reported to inhibit macrophage clearance of apoptotic cells and platelet aggregation [73], leading to the inhibition of apoptotic neutrophil clearance [107].

Targeting the AXL receptor domain directly is the leading strategy in this field, with a few engineered decoy receptors in development [108,109] and the most advanced Batiraxcept (formerly AVB-500) in the lead. Batiraxcept inhibits GAS6/AXL signalling in vivo and shows an 80-fold greater affinity to GAS6 than the natural receptor. Batiraxcept construct carries four point mutations (Asp87Gly, Val92Ala, Gly32Ser, and Gly127Arg), allowing the decoy receptor to trap the GAS6 ligand by both minor and major binding sites in a heterobivalent manner [110]. Moreover, Batiraxcept demonstrated a favourable safety profile in clinical trials and is now being tested for platinum-resistant ovarian cancer treatment in combination with paclitaxel in a phase III clinical trial (NCT04729608).

### 3.3. Low-Molecular-Weight Compounds Targeting ECD of TAMs

An alternative approach to inhibit TAMs with small non-biological compounds was also tested. RU-301 and RU-302 are low-molecular-weight (LMWs) compounds that bind to Ig1 domains of TAM extracellular domains, blocking ligand binding. Both compounds showed good inhibitory effects in low micromolar IC_50_s, with the activation of both TAMs being inhibited in cultured cancer cells and tumour growth in lung cancer xenograft models [111]. The inhibitory parameters might be lower than the tyrosine kinase inhibitors described in the next chapter, but RU-301 showed much higher specificity against TAM receptors. Kinase profiling revealed that RU-301 is much more specific and presents less off-target activities than R428, a strong AXL TKI with 14 nM IC_50_ against AXL [111]. AXL activity is also suppressed by a well-established anti-coagulant—warfarin. Low dosages of warfarin prevent the progression and spread of pancreatic cancer [112]. In this scenario, warfarin inhibits the activation of vitamin K by epoxide reductase complex 1 (VKORC1) and, by proxy, blocks the activation of GAS6, which is a vitamin K-dependent protein [113]. Consequently, an induced apoptosis of cancer cells was observed, and reduced migration, proliferation, and improved sensitivity to chemotherapy were revealed [112].

## 4. Kinase Domain: An Approach from the Inside

### Tyrosine Kinase Inhibitors

The intracellular kinase domain of TAM receptors is considered a very promising therapeutic target in cancer therapy. Kinase domains are very conservative in their structure, which allows the wide use of bioinformatic approaches in developing small-molecule inhibitors. The intracellular domains of TAM receptors share 54–59% sequence identity and very high (72–75%) sequence similarity [6,114]. The homology is also represented in the overall structure of TAM kinase domains, with very similar topography and global conformation.

Crystal structures of MERTK/AXL/TYRO3 kinase domains show that all share a consensus KW(I/L)A(I/L)ES sequence; however, MERTK and AXL are structurally closer to each other than to TYRO3 [115]. AXL and MERTK form very similar pockets in ATP-binding sites, while the TYRO3 kinase domain has a bigger pocket that accommodates larger molecules [115]. Crucial amino acids for ATP binding have been identified in MERTK and are conserved within TYRO3 and AXL. These crucial residues are as follows: Leu593, Gly594, Val601, Ala617, Lys619, Leu671, Pro672, Phe673, Met674, Asp678, Arg727, Asn728, Met730, and Asp741 in MERTK. There are also two important substitutions reported: Ile650Ala in TYRO3 and Ile650Met in AXL [115]. It has been shown that Ile650Met introduced in MERTK results in the formation of a mimic AXL active site, whereas substitution Ile650Ala does not mimic the TYRO3 catalytic site due to larger structural differences in the TYRO3 catalytic pocket, where Ile650A forms a unique subpocket near the ATP binding site [115,116]. All of these structural differences mean that bigger inhibitors bind better to TYRO3, while smaller ones have a lower affinity towards TYRO3, with low selectivity between MERTK and AXL due to the high similarity of the two domains. This effect is visible in the IC_50_ values of different TAM TKIs (Table 1). The computational analysis supports this notion, as a large inhibitor compound designed for MERTK/AXL presented a higher affinity towards TYRO3 than the kinases it was targeting [116]. These subtle differences can be exploited to improve selectivity between TAM family members, as an overall conserved structure of the kinase domain results in difficulties in creating the potent and selective inhibitors.

Typically, the kinase domain consists of a β-strand N-lobe, and an α-helical C-lobe connected with a hinge. Kinase domain (KD) activity is regulated by the conformational state of the aspartate–phenylalanine–glycine (DFG) motif in the hinge region. In its inactive state, also called DFG-out, phenylalanine disrupts the orientation of the aspartate, effectively inhibiting Mg^2+^ binding and sterically blocking the ATP binding site. Upon activation, a trans-autophosphorylation of tyrosine residues occurs with conformational changes leading to the reorientation of phenylalanine and positioning of aspartate for two magnesium ions coordination, opening the hinge into a DFG-in state [152]. This mechanism is exploited in small-molecule inhibitor design, as well as an amphiphilic environment of the kinase domain active site, with polar and nonpolar residue clusters within the pocket. The highly conserved kinase structure of the kinase domain makes it difficult to design and develop new small-molecule inhibitors that are both highly selective and potent [115]. Nevertheless, properly implemented drug design can use this specific topography to create an intricate network of interactions to design specific inhibitors, which is crucial in the development of inhibitors for so closely related proteins as TAM family RTKs.

As growing information is gathered on kinase domain structure and its conformational states, several strategies for KD inhibition have been proposed. Currently, there are six different classes of inhibitors, representing different approaches to kinase domain inhibition. Type I inhibitors interact with the ATP-binding site in the active DFG motif (DFG-in) state competitively with ATP. Type II inhibitors occupy the same area, but in a DFG-out state, keeping the kinase domain inactive [116]. Type I/II inhibitors are the most advanced TKIs in development, but are limited by highly conserved KD structure, which often results in the low selectivity and high toxicity of these inhibitors. Both classes are also susceptible to acquired drug resistance through mutations affecting the gatekeeper residues. New-generation type II inhibitors are designed to penetrate an allosteric pocket to overcome drug resistance [152]. Type III inhibitors target specific allosteric sites within the catalytic site, while type IV inhibitors target other allosteric sites. Type III/IV inhibitors are highly selective, as they target specific regulatory sites of enzymes, e.g., phosphorylation sites [153]. Type V inhibitors use protein scaffolds for bivalent binding of KDs’ active sites and other important sites, like the structural motifs or regulatory sites. Type V inhibitors are potent tools in research, but their applications in cancer treatment are limited by the large size of protein scaffolds, which restrict their availability to RTKs’ intracellular KD domains. Type VI inhibitors target active kinase sites and bonds in a covalent way. Different and stable binding lead to high potency and reduced toxicity in comparison to type I and II inhibitors [154,155]. Type VI inhibitors are also more resistant to acquired drug resistance via gatekeeping mutations within ATP-binding sites [155].

**Figure 2:**
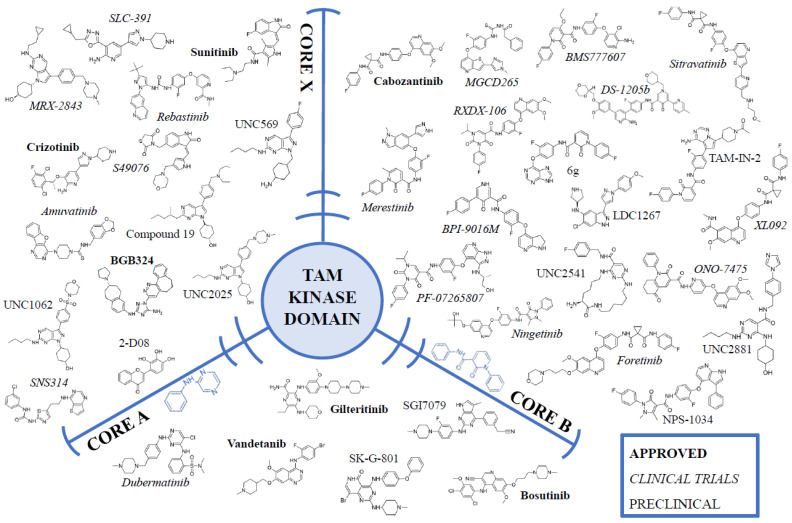
Different structures of TAM family kinase inhibitors. TAM receptors are divided into three groups, based on their base core structure: CORE-A compounds, with hydrogen bond acceptor–substituted phenyl group linked to a hinge-binding heterocycle with a solubilising group; CORE-B compounds, with heterocycle (hinge-binding) optionally ortho-fluoro phenyl group (binding DFG motif), 2–4 hydrogen donors/acceptors, and a phenyl group (or para-fluoro phenyl) (binding to allosteric hydrophobic pockets); and CORE-X compounds, that cannot be clearly assigned to either -A, or -B groups, as they present only partial structural similarity to other cores, or present completely different lead structures. Compounds approved for treatment are presented in bold, compounds currently in clinical trials are presented in italics, and compounds that did not enter the clinical phase are presented in plain text.

In the case of the TAM family, the TKIs of type I and type II are the most prominent and well-studied [156]. These compounds can be aggregated in two distinct structural groups with similar cores, and some non-conventional compounds. First, the CORE-A group consists of smaller compounds, with a hydrogen bond acceptor–substituted phenyl group linked to a hinge-binding heterocycle with a solubilising group (Figure 2). These compounds (SGI7079, bosutinib, gilteritinib, dubermatinib, vandetanib) are all type I inhibitors and were often designed primarily for AXL and present high inhibition of AXL in the low-nM range, with similar inhibition levels of MERTK (Table 1). CORE-A inhibitors are very successful, with three of these compounds (gilteritinib, bosutinib, and vandetanib) developed beyond the preclinical stage with successful clinical trials, and are now approved for treatment in different types of cancer [157].

The second group of compounds, i.e., CORE-B, presents a more complex structure: heterocycle (hinge-binding) optionally ortho-fluoro phenyl group (DFG-binding), 2–4 hydrogen donors/acceptors, and a phenyl group (or para-fluoro phenyl) that binds to allosteric hydrophobic pockets (Figure 2). This scheme is prominent among type II inhibitors, as these compounds are generally bigger and cannot properly dock ATP-binding sites in a DFG-in state [158,159]. This class is developing rapidly, with several compounds in various clinical trial phases (BMS777607, DS-1205c, foretinib, MGCD265, merestinib, ONO-7475, PF-07265807, sitravatinib) and one, cabozantinib, approved for treatment. Several more compounds are yet to be tested for efficacy in clinical trials (Table 1).

As more TAM TKIs are developed, new compounds are introduced, presenting partial structural similarity to previously described cores, -A and -B (rebastinib, amuvatinib, SNS314), or present completely different lead structures (crizotinib, sunitinib, S49076, UN1062, UNC2025). Those compounds are mostly type I inhibitors, the exception being type II rebastinib. These unique cores open new ways for the rational design of TKIs [158,159], as most successful compounds here were designed for different kinases (e.g., crizotinib for ALK/c-Met/ROS1, foretinib for MET/VEGFRs and suntinib for VEGFR2/PDGFRs), but their robust cores are potent against other receptor kinases, TAMs included (Figure 2).

## 5. Battles for the Future

### 5.1. Selectivity of TAM Family Inhibitors

As discussed above, a rational approach to developing selective inhibitors within the TAM family is challenging, due to the high structural homology around the active catalytic site. In particular, AXL and MERTK are similar—their ATP-binding site is smaller compared to the more open TYRO3 pocket [115]. Consequently, it is easier to obtain dual AXL/MERTK inhibitors that show selectivity over TYRO3 [115]. When acting on more than one target, there should always be broad consideration of what this might entail—what the concerns might be versus the potential benefits.

Importantly, targeting closely related kinases may involve resistance caused by so-called bypass signalling [77,78]. For example, AXL can cause resistance to MERTK and MERTK can cause resistance to AXL [160]. Given this consideration, in some cases, the simultaneous targeting of both of these kinases may be more effective than the selective inhibition of either kinase. McDaniel et al. showed that the expression of MERTK kinase is increased after treatment with AXL inhibitors in cancer cell lines and patient-derived xenografts [160]. MERTK inhibition resulted in increased sensitivity of head and neck squamous cell carcinoma (HNSCC), triple-negative breast cancer (TNBC), and non-small-cell lung carcinoma (NSCLC) cell lines to AXL inhibition. When both kinases, AXL and MER, were targeted, it caused more robust inhibition of downstream signalling and impaired tumour cell expansion in vitro, as well as reduced tumour growth in vivo [160].

In NSCLC, AXL and MER may have overlapping and complementary roles; furthermore, their role in resistance to therapies and co-occurrence in the tumour microenvironment may outweigh the benefits that the development of such a concept may bring [17]. The inhibition of more than one kinase from the TAM family may be beneficial in some aspects, as described above, but on the other hand, it may also result in increased adverse effects, i.e., inflammation. AXL is often overexpressed in TNBC with a mesenchymal phenotype and in some colon cancer subtypes [161,162,163]. In contrast, MERTK kinase seems to be more abundantly expressed in acute lymphoblastic leukaemias, therefore a selective MERTK inhibition could be an interesting therapeutic strategy in this context [164,165,166,167].

### 5.2. Combination Therapy

Combination therapies are implemented in cancer treatment for several reasons. First, they increase the effectiveness of the therapy and improve treatment outcomes, particularly when synergistic anticancer effects are achieved. Second, reduced emerging resistance to therapy is observed, which is a key and limiting problem. Studies are being conducted on combining therapies targeting TAM receptors and classical therapies such as radio- or chemotherapy, as well as targeted therapies and immune-checkpoint inhibitors. There is a growing body of knowledge linking TAM kinases to resistance to chemotherapy and radiotherapy in both solid and hematologic malignancies [168,169]. Moreover, in the case of therapies targeting, e.g., EGFR in NSCLC, TAM receptors are known to mediate bypass signalling, resulting in resistance to EGFR inhibitors such as erlotinib, gefitinib, and osimertinib [36,170,171,172]. Moreover, kinases of this family, through their physiological functions in the immune system, can also modulate resistance [50,173,174]. Several TAM inhibitors that are still in development, besides their direct antitumour activity, are known for their immunomodulatory effects on the tumour microenvironment and enhancement of the antitumour immunity [133,141]. Therefore, there are ongoing clinical trials combining TAM TKIs and immune checkpoint inhibitors such as pembrolizumab and nivolumab [175,176,177,178]. Combining TAM-targeted inhibitors with classical, targeted, or immunotherapies seems reasonable to obtain more effective anticancer treatment strategies.

### 5.3. Biomarkers

Despite the frequent overexpression of TAM proteins in cancer, genetic mutations or gene amplification are rare [77,179]. Due to the lack of confirmed activating mutations or amplifications that could be a suitable biomarker of therapeutic response, extensive work is being done to find robust biomarkers. Finding the appropriate biomarkers is one of the challenges in developing therapies targeting TAM kinases that could lead to clinically successful treatment strategies. Other options include the expression of the TAM family proteins, their phosphorylation and activation status. Moreover, the level of ligands (GAS6 in particular) and soluble forms of the receptors are taken into account as potential biomarkers [179,180]. What is more, it may be a challenge to find a universal biomarker for different cancer types, which is related to tissue-dependent signalling and ligand levels in specific cancer types [95,181]. Currently, in the ongoing clinical trials, i.a., AXL protein levels and AXL phosphorylation status are being analysed [182].

### 5.4. Potential Concerns

The development of anticancer therapy targeting TAM family receptors requires the consideration of their expression and function in the normal cells of the body. It is worth mentioning that MERTK and AXL kinases are expressed in immune cells such as macrophages, NK cells, and dendritic cells [47,64,183]. Studies of single, double, and triple knockouts of TAM receptors in mice have demonstrated, e.g., inflammation and autoimmunity, with more severe dysfunctions when targeting more than one kinase [39,43,44]. Moreover, as mentioned above, in the case of less selective TAM inhibitors, structurally-related receptors may also be inhibited, leading to increased off-targets and toxicities. TAM receptors promote tissue-specific macrophage polarisation into a pro-tumour M2-like phenotype as AXL and TYRO3 regulate phagocytosis in dendritic cells, whereas MERTK do so in the thymus and retina [64,156,184]. Retinal toxicity is observed under MERTK inhibition and is one of the main concerns in the development of TAM inhibitors [185,186]

As other reports suggest, the activity and downstream signalling of TAM receptors is also regulated by homodimerisation, and this intricate web of interactions and relations makes it difficult to create an inhibitor that is selective, effective, and does not deregulate other signalling pathways [64]. On the other hand, in the case of single-agent anticancer therapy, the emergence of resistance is a well-known and inevitable phenomenon. Upon AXL inhibition, MERTK is upregulated in several cancer models and constitutes one of many mechanisms of drug resistance build-up [160]. So far, mechanisms of resistance to TAM-targeted therapies have not been extensively studied, besides the abovementioned preclinical evaluation of MERTK and AXL’s mutual impact on resistance.

## 6. Future Directions

In summarising the emerging data and collected information, it is becoming clear that the further development of therapies targeting TAM kinases is necessary. It is worth emphasising that small molecular inhibitors are effective, but often highly toxic. On the other hand, antibodies are much more selective and less toxic, but so far do not show as strong a therapeutic effect as small molecular inhibitors. For the further development of TAM-targeted therapies, it would be crucial to find biomarkers stratifying patients to predict which of them may present the greatest therapeutic response. As the effect of targeting TAM kinases, especially AXL and MERTK, is known to increase the effectiveness of other therapies such as chemotherapy, immunotherapy, radiotherapy, and targeted therapies, it is crucial to gather the most in-depth data on the possible and most promising clinical combinations. In addition, further studies should be also implemented to reveal potential toxicities, relevant clinical regimens, therapeutic strategies, and possible bypass mechanisms and drug resistance.

## 7. Conclusions

In this review, we provide a comprehensive picture of the current status of the TAM receptor inhibition strategies in solid tumour therapy. We present how structural differences between TAM receptors can be exploited to inhibit their activity, with antibodies in various formats, antibody–drug conjugates, protein decoys, and small compounds targeting their extracellular domains and on the other side, the wide array of tyrosine kinase inhibitors directly blocking signal transduction via the intracellular kinase domains.

The great complexity of TAM receptors’ activation mechanisms and their involvement in carcinogenesis make it difficult to develop a drug that is effective, specific, and safe for patients. Hence, we need to further understand how the different structures of low-molecular-weight compounds and antibody formats define their properties and characteristics to overcome acquired drug resistance and make these drugs even more potent in the future. Here, we do not present one approach as better than the other, as we firmly believe that every way to battle cancer is the right way in this never-ending war on cancer.

## Figures and Tables

**Table 1:** TAM family kinase domain inhibitors in cancer therapy.

Inhibitor	Status ^1^	Core	Type	Inhibitory Parameters	References
Bosutinib; SKI-606; PF5208763; Bosulif	APPROVED	A	I	AXL IC_50_ = 174 nM MERTK IC_50_ = 110 nM TYRO3 IC_50_ = 61 nM	[117,118,119]
Gilteritinib; ASP2215; Xospata	APPROVED	A	I	AXL IC_50_ = 0.73 nM MERTK IC_50_ = 5 nM	[120]
Vandetanib; ZD6474; Caprelsa	APPROVED	A	I	AXL IC_50_ = 250 nM MERTK IC_50_ = 1400 nM TYRO3 IC_50_ = 93 nM	[119]
Cabozantinib; XL 184; BMS-907351; Cabometyx	APPROVED	B	II	AXL IC_50_ = 7 nM	[121]
BGB324; R428; Bemcentinib	APPROVED	X	I	AXL IC_50_ = 14 nM	[122]
Crizotinib PF-02341066; Xalkori	APPROVED	X	I	AXL IC_50_ = 294 nM	[123]
Sunitinib; SU 11248; Sutent	APPROVED	X	I	AXL IC_50_ = 9 nM	[124]
TP-0903; Dubermatinib	CLINICAL TRIALS NCT04518345	A	I	AXL IC_50_ = 27 nM	[125]
BMS777607; ASLAN002	CLINICAL TRIALS NCT01721148 NCT00605618	B	II	AXL IC_50_ = 1.1 nM MERTK IC_50_ = 14 nM TYRO3 IC_50_ = 4.3 nM	[126]
BPI-9016M	CLINICAL TRIALS NCT02929290 NCT02478866	B	II	AXL IC_50_ = 9 nM	[127]
DS-1205b/c	CLINICAL TRIALS NCT03599518 NCT03255083 (TERMINATED)	B	II	AXL IC50 = 1.3 nM MERTK IC50 = 63 nM	[128]
Foretinib; XL880; GSK1363089	CLINICAL TRIALS NCT00920192 NCT01147484 NCT01138384 NCT00742131 NCT00725764 NCT00726323 NCT00725712 NCT00743067 NCT01068587	B	II	AXL IC_50_ = 11 nM	[129]
Merestinib; LY2801653	CLINICAL TRIALS NCT03125239 NCT03027284 NCT02779738 NCT02745769	B	II	AXL IC_50_ = 2 nM MERTK IC_50_ = 10 nM	[125,130]
MGCD265; Glesatinib	CLINICAL TRIALS NCT02954991	B	II	n/a	
Ningetinib; CT053PTSA	CLINICAL TRIALS NCT04577703 NCT03758287	B	II	AXL IC_50_ < 1.0 nM	[131]
ONO-7475	CLINICAL TRIALS NCT03176277	B	II	AXL IC_50_ = 0.7 nM MERTK IC_50_ = 1 nM TYRO3 IC_50_ = 1.9 nM	[132]
PF-07265807; ARRY-067; PF-5807	CLINICAL TRIALS NCT04458259	B	II	n/a	
RXDX-106; CEP-40783	CLINICAL TRIALS NCT03454243 (TERMINATED)	B	II	AXL IC_50_ = 0.31 nM MERTK IC_50_ = 1.89 nM TYRO3 IC_50_ = 3.5 nM	[133]
Sitravatinib; MGCD516	CLINICAL TRIALS NCT04123704 NCT03575598 NCT04472650 NCT04772612 NCT04921358 NCT04727996 NCT04800614 NCT04904302 NCT04925986 NCT05176925 NCT05104801 NCT05255276 NCT04887194 NCT04734262 NCT02954991 NCT03606174 NCT04887870	B	II	AXL IC_50_ = 1.5 nM MERTK IC_50_ = 2 nM	[134]
XL092	CLINICAL TRIALS NCT03845166 NCT05176483	B	II	AXL IC_50_ = 3.4 nM MERTK IC_50_ = 7.2 nM	[135]
Amuvatinib; MP470	CLINICAL TRIALS NCT01357395 NCT00894894 NCT00881166	X	I	AXL IC_50_ = 10 nM	[57]
MRX-2843; UNC2371	CLINICAL TRIALS NCT03510104 NCT04762199 NCT04872478	X	I	AXL IC_50_ = 15 nM MERTK IC_50_ = 1.3 nM TYRO3 IC_50_ = 17 nM	[136]
S49076	CLINICAL TRIALS ISRCTN11619481	X	I	AXL IC_50_ = 7 nM MERTK IC_50_ = 2 nM	[137]
SNS314	CLINICAL TRIALS NCT00519662	X	I	AXL IC_50_ = 84 nM	[138]
Rebastinib; DCC-2036	CLINICAL TRIALS NCT00827138	X	II	AXL IC_50_ = 42 nM	[139]
SLC-391	CLINICAL TRIALS NCT05278845 NCT03990454	X	n/a	AXL IC_50_ = 9.6 nM MERTK IC_50_ = 42.3 nM TYRO3 IC_50_ = 44 nM	[140]
INCB081776	CLINICAL TRIALS NCT03522142	n/a	n/a	AXL IC_50_ = 0.61 nM MERTK IC_50_ = 3.17 nM TYRO3 IC_50_ = 101 nM	[141]
Q702	CLINICAL TRIALS NCT04648254	n/a	n/a	n/a	
SGI7079	PRECLINICAL	A	I	AXL IC_50_ = 58 nM	[142]
SK-G-801; G-801	PRECLINICAL	A	I	AXL IC_50_ = 20 nM	[143]
6g; purine analogue of BMS777607	PRECLINICAL	B	II	AXL IC_50_ = 39 nM MERTK IC_50_ = 42 nM TYRO3 IC_50_ = 65 nM	[144]
LDC1267	PRECLINICAL	B	II	AXL IC_50_ = 29 nM MERTK IC_50_ = 5 nM TYRO3 IC_50_ = 8 nM	[47]
NPS-1034	PRECLINICAL	B	II	AXL IC_50_ = 10.3 nM	[145]
TAM-IN-2	PRECLINICAL	B	II	n/a	
UNC2541	PRECLINICAL	B	II	MERTK IC_50_ = 4.4 nM	[146]
UNC2881	PRECLINICAL	B	II	AXL IC_50_ = 360 nM MERTK IC_50_ = 4.3 nM TYRO3 IC_50_ = 250 nM	[147]
2-D08	PRECLINICAL	X	I	AXL IC_50_ = 0.49 nM	[148]
UNC1062	PRECLINICAL	X	I	AXL IC_50_ = 85 nM MERTK IC_50_ = 1.1 nM TYRO3 IC_50_ = 60 nM	[23,149]
UNC2025	PRECLINICAL	X	I	AXL IC_50_ = 14 nM MERTK IC_50_ = 0.7 nM TYRO3 IC_50_ = 18 nM	[150]
Compound 19	PRECLINICAL	X	n/a	TYRO3 IC_50_ = 10 nM	[151]

Abbreviations: n/a, not available; IC_50_, half-maximal inhibitory concentration. ^1^ The table lists NCT/ISRCTN clinical trials numbers only for completed or recruiting trials. For RXDX-106, the NCT number is given for terminated study (sponsor’s decision) as it was the only clinical trial for this compound.
